# Clinical data required for the approval of pediatric pharmaceuticals in Japan

**DOI:** 10.1186/s12887-025-05646-0

**Published:** 2025-04-14

**Authors:** Hisamitsu Haigo, Kenji Matsuda, Mayumi Shikano

**Affiliations:** Graduate School of Pharmaceutical Sciences, Tokyo University of Science, Tokyo, Japan

**Keywords:** Pediatric, Drug development, Drug lag, Data package, Extrapolation

## Abstract

**Background:**

In Japan, the number of pharmaceuticals with pediatric indications is low, and some are approved only in Europe or the USA but not in Japan. As the approval review report by the Japanese health authority contains any detailed items considered for drug approval, this study aimed to analyze the review reports and elucidate data types that facilitate the approval of pediatric drugs in Japan.

**Methods:**

We identified products approved in Japan, extracted relevant product- and review-related information, and summarized the characteristics of pediatric drug clinical data and drug approval procedures.

**Results:**

Among 625 products (approved 04/2019–02/2024), 171 with pediatric indications were analyzed. The approval review considered orphan drug designation for 56 products, public knowledge-based application for 16 products, mandatory post-marketing surveillance for 42 products, and investigator-initiated studies for 11 products.

For only 10 products, confirmatory studies were completed exclusively in Japanese children. Among the other 161 products, extrapolation from non-Japanese children and Japanese adults and/or older children was discussed for 93 and 100 products, respectively. Extrapolation-based reviews focused on ethnic and population factors and consistency of exposure dose, efficacy, and safety.

Statistical confirmation is not always necessary for approval. Administrative incentives are often applied, including for orphan drugs and Sakigake designation and public knowledge-based applications.

**Conclusions:**

The appropriateness or sufficiency of the clinical data package can refer to the PMDA. By considering joining a multinational study and determining the required number of Japanese patients, a path toward the approval of pediatric drugs in Japan can be identified.

## Introduction

In Japan, the number of pharmaceuticals with pediatric indications is smaller than those for adults, with limited scope [1]. Overall, 60–70% of pediatric drugs are used off-label [2], primarily due to slow pediatric drug development. The smaller pediatric population results in lower revenue for pharmaceutical companies. Additional factors reducing profits include the need for multiple drug variations to accommodate children's growth [3], required costs for small markets, difficulty in patient recruitment [4], and the necessity of parental cooperation and special considerations in clinical trials.

In Europe and the USA, mandatory measures promote pediatric drug development [6, 7]. The pharmaceutical company agrees on the Pediatric Study Plan and the Pediatric Investigational Plan with the U.S. Food and Drug Administration and the European Medicines Agency, respectively, during adult drug development. In Japan, a similar process was recently launched but is not mandatory [5]; however, voluntary measures have been incentivized, including prolonged exclusive marketing periods and premium prices when pediatric indications are approved [1]. However, many drugs approved in Europe or the USA remain unapproved in Japan, contributing to a problematic "drug lag or loss" [4, 8, 9].

Despite this, ~ 20–30% of newly approved drugs in Japan each year are for pediatrics [1]. This study analyzed clinical data packages and reviewed the development history of these products to identify strategies for pharmaceutical companies seeking pediatric indications in Japan, groups planning investigator-initiated studies (IIS), and companies not previously considering pediatric drug development. These insights may help advance pediatric drug development in Japan.

## Methods

### Study type

A retrospective study was conducted based on the publicized drug approval review reports by the Japanese health authority. The clinical studies included in the review report were in compliance with the ICH GCP and approved by the local ethics committee or institutional review board (IRB), and these approvals were inspected by the health authorities of Japan or other countries.

### Identification of Approved Pediatric Drugs

The Pharmaceuticals and Medical Devices Agency (PMDA) provides a list of approved pharmaceutical products [10]. Based on this list, excluding generic drugs, we identified products newly approved in Japan between April 2019 and February 2024.

The PMDA discloses the latest information regarding approved drugs [11], including package inserts, interview forms, risk management plans, and usage guidelines, as well as review reports and Common Technical Documents (CTDs) Module 2. Among newly approved products, we identified those with regulatory approval discussions for further analysis. Products without pediatric indications and biosimilars were excluded.

### Extraction of Relevant Information on Identified Drugs

Information regarding targeted products listed in Table 1 was extracted from the PMDA review reports and package inserts. PMDA review reports include the perspectives of both applicants and the PMDA; however, only the PMDA views were extracted. Applicants’ comments were included if in agreement with the PMDA.

**Table 1:** Extracted information

From PMDA review report	From package insert
Approval date Non-proprietary name Brand name Modality Route of administration Approval classification ^a^ Administrative incentives (see Table 2) Pediatric clinical studies referred for PMDA review ^b^ Phase Region (Japan, countries apart from Japan, multi-national studies including Japan) Number of patients (Japanese, non-Japanese) Whether a statistical confirmatory study was conducted or not Whether an investigator-initiated study was conducted or not Discussion points considered for pediatric indications Conditions for approval	Indication Target age Dosage and administration for pediatric patients Precautional statements in the package insert Sect. 9.7 ^c^

^a^Approval classification; Specifies whether an approval is for a drug with a new active ingredient, an additional indication, a new dose, a new dosage form, or a new route of administration for an existing product

^b^Information on clinical studies included in the review was extracted from Sect. 7 of the PMDA review report. If unavailable, it was sourced from the CTD Module 2.7 disclosed on the PMDA website

^c^Japanese package inserts specify precautions for children in this section, categorized by age group

*CTD* Common Technical Document, and *PMDA* Pharmaceuticals and Medical Devices Agency

**Table 2:** Administrative incentives

Measures	Contents
Orphan drug designation	Designated in Japan when all three conditions (basically less than 50,000 patients, medical necessity, and possibility to be developed in Japan) are met
Pioneer drug designation Sakigake	With the goal of providing patients with the earliest available state-of-the-art therapeutic agents, drugs that are developed in Japan earlier than in the rest of the world, have expected significant efficacy, and are registered in the early clinical stage receive priority handling in consultation and review related to regulatory approval
Public knowledge-based application	A system for drugs is already approved in Japan to provide approval without having conducted some or all clinical trials in Japan, if there is a scientific basis like approval in a foreign country, experience of use, or a rationale-providing paper
Recommendation in the “Review Meeting of Unapproved Drugs and Off-label Drugs with High Medical Needs”	For drug indications approved in Europe and the USA but not in Japan, drug use requested by academic societies can be approved if the meeting body recognizes the necessity and recommends the drug for approval
Public knowledge-based application after pre-evaluation	Products that have been submitted for public knowledge-based application after being evaluated by the abovementioned committee
Priority review	A system that prioritizes the review of drugs for approval that meet certain standards
Pre-evaluation	Preliminary evaluation based on available data on quality, nonclinical (toxicology, pharmacology, and pharmacokinetics), and clinical studies (phase 1, 2, and 3) in the pre-submission development stage. This consultation system precedes the approval review process. As it allows the extraction and resolution of issues at each development stage before the application, the approval review period is shortened
Conditional early approval system	A drug can be approved if it targets a serious disease, unavailability of effective therapy for the disease, the number of patients is small, and it is difficult or takes a long time to conduct a clinical trial in Japan. A certain level of efficacy and safety without confirmatory studies available at the time of application has to be ascertained, and the drug is approved on the condition that post marketing studies reaffirm the efficacy and safety
Drugs for a specific purpose	Designated by the Ministry of Health, Labor and Welfare based on the assessments of the “Pharmaceutical Affairs and Food Sanitation Council” if the demand for the use is not markedly met or the need for medical treatment is particularly high. It aims to contribute to the promotion of research and development of drugs for which medical needs are not markedly met, such as the lack of established doses and administration routes for children
COVID- 19-related priority review	A now terminated system for priority review of drugs, quasi-drugs, medical devices, in vitro diagnostics, and regenerative medicine for COVID- 19 infections or related symptoms
Special approval	An early approval system for drugs which are available in the US or EU and required urgent use to prevent the spread of diseases that may seriously affect the lives and health of the public and for which no other appropriate method is available
Mandatory post-marketing surveillance	Some new drugs are approved on the condition that post-marketing surveillance is conducted in all patients. It is often required when limited clinical trial data are available at the time of approval such as for orphan drugs or drugs for which concern exists about the occurrence of serious adverse events

### Aggregation by Background Factors

**Fig. 1:**
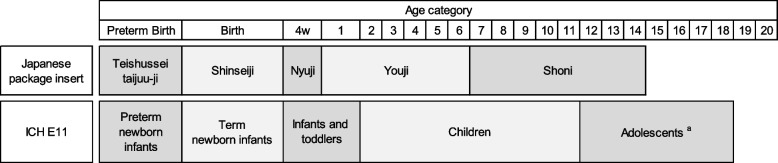
Differences in age categories between Japanese package inserts and the international classification system (ICH E11). a 12–16 is possibly a case depending on region. *ICH* Common Technical Document, *PMDA* Pharmaceuticals, and Medical Devices Agency

The analyzed products were categorized by approval classification, modality, administration route, and target age according to the information in the package insert. The age categories in Japanese package inserts, differing from those of the international classification (Fig. 1), were used to tabulate the number of approved products by age group.

### Analysis of “Pediatric Clinical Study to Be Reviewed” and “Discussion Points for Approval in Children”

In the PMDA review report, “Discussion points to give approval for children” is provided as a text description. For analysis, we extracted and tabulated the following key points:

- Highly urgent for approval.
- No alternative treatments available.
- Pharmacokinetics in Japanese children can be predicted from non-Japanese children.
- Pharmacokinetic/pharmacodynamic relationships in Japanese children can be predicted from non-Japanese children.
- Data are extrapolated from adults and/or older children.
- Data are extrapolated from non-Japanese children.
- Pediatric use is recommended in guidelines or theoretically established.
- Approved in countries other than Japan.
- Already used for treatment.

## Results

### Identified Drugs

**Fig. 2:**
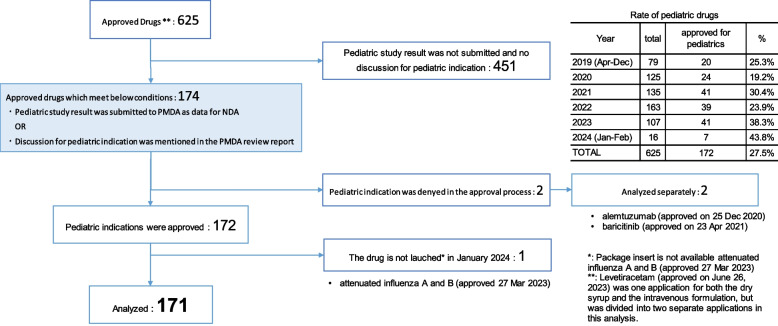
Flowchart of the analyzed products

Based on the PMDA’s list of approved products, 625 products were approved between April 2019 and February 2024 (Fig. 2). Data on pediatric clinical studies or pediatric indications for review were submitted for 174 products, which were discussed in the PMDA review report. Of these, 171 were included in the analysis, excluding:

- Two products for which pediatric indications were not approved

- One product that had not been launched as of April 2024.

Thus, we analyzed 27.5% (171/625) of all approved drugs, accounting for 20–40% of annual approvals.

The two excluded products were alemtuzumab and baricitinib, for which new indications for allogeneic hematopoietic stem cell transplantation and for severe acute respiratory syndrome coronavirus 2 (SARS-CoV- 2) infection were approved on December 25, 2020, and April 23, 2021, respectively. The applicant provided a reference for clinical studies investigating the clinical usefulness of alemtuzumab as a pretreatment drug [12, 13]; however, the indication was approved only for adults, as clinical studies were unavailable to evaluate the efficacy and safety of an alemtuzumab administration of 0.16 mg/kg in Japanese pediatric patients. For baricitinib, the applicant did not submit pediatric data. Pediatric indications were claimed based on emergency use authorization in the USA. However, the indication was only approved for adults owing to the unavailability of pediatric studies, and the efficacy and safety of drugs in children could not be confirmed.

Attenuated virulent influenza viruses (types A and B) were approved on March 27, 2023, and included in the “Rate of pediatric drugs” table. However, they were excluded from the analysis, as the review report was not published as of February 2024.

### Aggregation by Background Factors

**Fig. 3:**
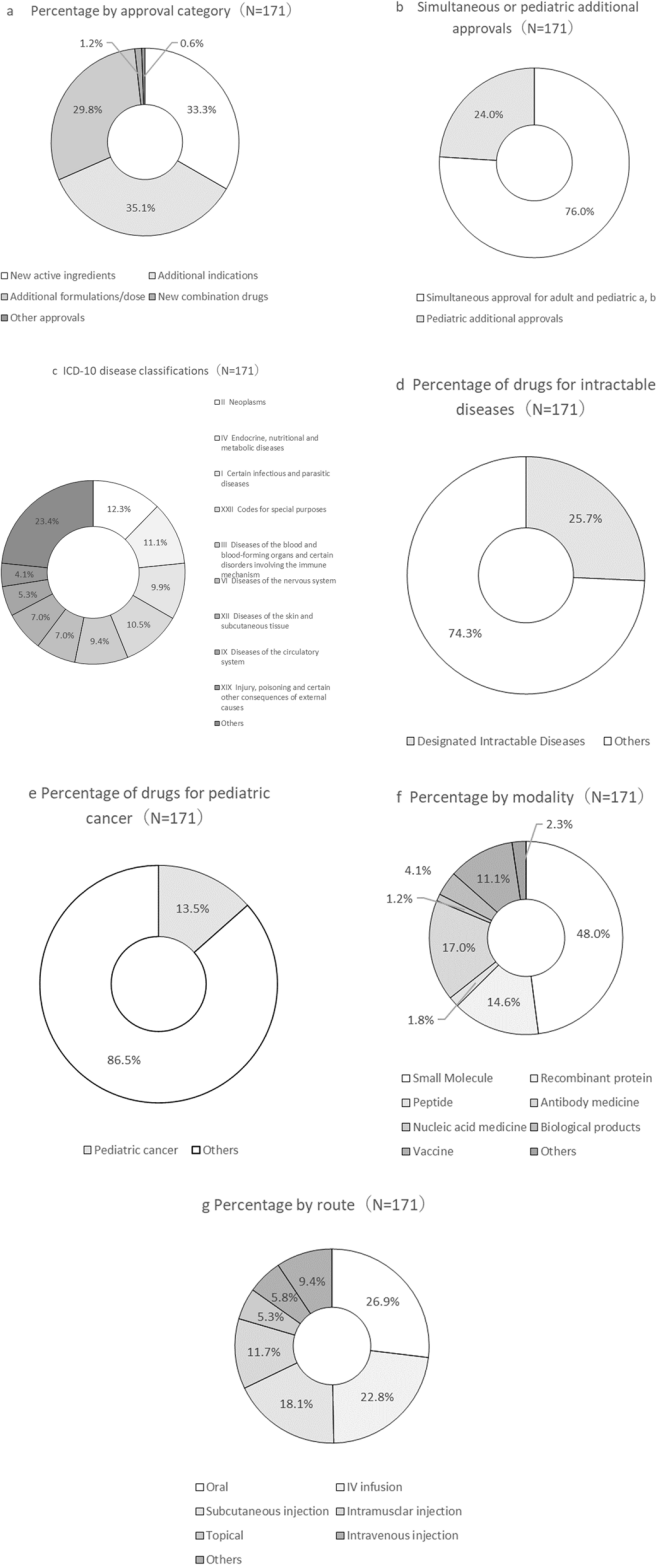
Characteristics of analyzed products. (a) Addition of new doses, dosage forms, and routes of administration for previously approved indications. (b) Two products were approved exclusively for children, not for adults (ranibizumab for retinopathy of prematurity, approved on November 22, 2019; valganciclovir hydrochloride for symptomatic congenital cytomegalovirus infection, approved on March 27, 2023)

**Fig. 4:**
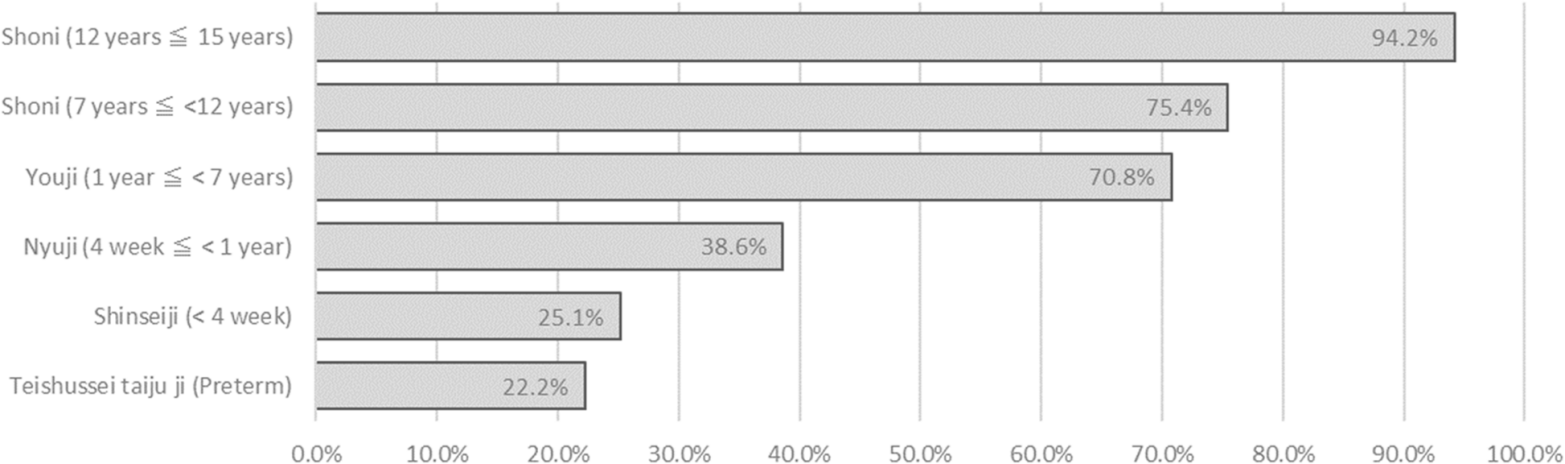
Percentage of approved products by age category

Figure 3 provides background information on the 171 products with pediatric indications. In addition to products for new active ingredients, approvals included new pediatric indications, doses, dosage forms, and administration routes. Most products were simultaneously approved for both children and adults. Drugs for intractable diseases and pediatric cancer are listed in Tables 8 and 9, respectively. When analyzing by age, most products were approved for children aged ≥ 12 years, with the number decreasing as the target age group became younger (Fig. 4).

### Extracted Discussion Points

In total, 32.7% (56/171) were designated as orphan drugs. Of these, 4 and 12 were public knowledge-based application products and public knowledge-based applications after pre-evaluation, respectively, totaling 9.4% (16/171). Moreover, 9.4% (16/171) were deliberated by the “Review Meeting of Unapproved Drugs and Off-Label Drugs with High Medical Needs.” Due to the COVID- 19 pandemic during the study period, vaccines and therapeutic agents for SARS-CoV- 2 infection have been approved using the “Special approval system” (9.9%, 17/171) and “COVID- 19-related priority review system” (4.1%, 7/171). For 24.6% (42/171) of products, all-patient surveys were requested in post-marketing surveillance (PMS). Among these, only 12 were evaluated in confirmatory clinical studies before application, and the remaining 30 products were approved without confirmatory studies.

The high urgency of approval was mentioned in the PMDA review report for 25.1% (43/171) of products, with a particularly high frequency among pediatric anticancer agents (34.8%, 8/23). For pediatric anticancer agents, a high frequency (39.1%, 9/23) of discussions referenced treatment guidelines and literature. IIS results were submitted for 6.4% (11/171) of products, including four for pediatric cancer. Among the products with IIS, confirmatory studies were conducted in only two products, including cyclosporin (for the acute phase of Kawasaki disease, approved on February 21, 2020) and valganciclovir (for symptomatic congenital cytomegalovirus infection, approved on March 27, 2023).

### Pediatric Studies

Figure 6a indicates the status of pediatric studies. 18.1% (31/171) were approved based only on Japanese clinical studies, 21.1% (36/171) included both Japanese and non-Japanese clinical studies, 26.9% (46/171) were approved based on multinational studies including Japan, and 14.6% (25/171) were approved based on non-Japanese studies. Products approved without any pediatric clinical trial accounted for 19.3% (33/171). Among the 33 products approved without pediatric clinical trials, 18 cases had already been approved for adults, or extrapolations from adult clinical studies were considered.

Confirmatory studies were submitted for 39.8% (68/171) of products (Fig. 6f). Among them, 16 (Table 3), 27, and 25 products had confirmatory studies conducted only in Japan, multinational studies including Japan, and non-Japanese studies, respectively. For orphan drugs and drugs for designated intractable diseases, the implementation rates of pediatric clinical trials were notably high (Fig. 6c and d). The implementation rate of pediatric clinical trials for anti-cancer products was relatively low, at 65.2% (15/23). Among these, only one confirmatory study was conducted for the recombinant 9-valent human papillomavirus-like particle vaccine (approved on July 21, 2020). Thirty-one products were approved based solely on Japanese clinical trials.

**Table 3:** Products approved based on Japanese pediatric confirmatory study

Indication	Generic name	Approval category	Age range in clinical study	Number of patients in studies
Acute phase of Kawasaki disease	Cyclosporine	Additional indication Additional dose	> 4 months	86
Difficulty of falling asleep associated with childhood neurodevelopmental disorders	Melatonin	New active ingredients	2–15 years	322
Treatment of influenza A or B virus infection	Laninamivir octanoic acid ester hydrate	Additional formulation for already approved indication	≤ 18 years	265
Atopic dermatitis	Delgocitinib	Additional dose and Additional formulation for already approved indication	2–15 years	137
Schizophrenia	Blonanserin	Additional dose for already approved indication	12–18 years	104
Disease prevention caused by human papillomavirus infection	Recombinant adsorbed 9-valent human papillomavirus virus-like particle vaccine (yeast origin)	New active ingredients	≥ 9 years	100
Atopic dermatitis	Difamilast	New active ingredients	2–14 years	417
Prevention of pertussis, diphtheria, tetanus, acute poliomyelitis, and *Haemophilus influenzae* type b infections	Bordetella pertussis protective antigen Diphtheria toxoid Tetanus toxoid Inactivated poliovirus types 1–3 (Sabin strain) *H. influenzae* type b oligosaccharide-CRM197 conjugate	New active ingredients New active combination	2–43 months	670
Short stature in SHOX anomaly without epiphyseal line closure	Somatropin (genetic recombinant)	New indication	3–10 years and 5 months	19
Prevention of infections caused by pertussis, diphtheria, tetanus, acute gray meningitis, and *H. influenzae* type b	Bordetella pertussis protective antigen Diphtheria toxoid Tetanus toxoid Inactivated poliovirus types 1–3 (Sabin strain) *H. influenzae* type b conjugate vaccine	New active ingredients New active combination	2–59 months	704

Among these, 16 included confirmatory studies (Table 3). Of these, 10 products had confirmatory studies conducted exclusively in pediatric populations, whereas the remaining six products involved both children and adults. The other 15 products were approved based on open-label or uncontrolled studies in Japan (Table 4).

**Table 4:** Products approved based on Japanese confirmatory study involving pediatric patients and adults

Indications	Generic name	Approval category	Age range in clinical study	Number of included patients
< Indicated species > Azithromycin susceptible: *Staphylococcus spp., Streptococcus spp.,* *S. pneumoniae, Corynebacterium spp., H. influenzae,* Acne spp. < Indicated symptoms > conjunctivitis, blepharitis, hordeolum, dacryocystitis	Azithromycin hydrate	Additional dose route for already approved indication	7–18 years	24 children
Seasonal allergic rhinitis (limited to patients with severe or most severe symptoms and insufficient response to existing treatments)	Omalizumab (genetic recombinant)	Additional indication Additional dose	12–14 years	4 children
Eczema/dermatitis of the head	Clobetasol propionate	New indication	12–14 years	7 children
Pruritus associated with atopic dermatitis (limited to patients with inadequate response to existing treatments)	Nemolizumab (genetic recombinant)	New active ingredients	13–18 years	10 children
Primary palmar hyperhidrosis	Oxybutynin hydrochloride	Additional dose for already approved indication	12–17 years	19 children
Adjunct to wrapping nail correction	Acetylcysteine	New indications New route	Age and number of children not specified	

### Extrapolation from Studies

Table 5 presents the number of products for which extrapolation was discussed. Extrapolations from non-Japanese children and Japanese adults and/or older children were considered for 93 and 100 products, respectively. In contrast, extrapolation was not discussed for 28 products.

**Table 5:** Extrapolation from non-Japanese pediatric studies and studies in both Japanese adults and/or older children

	Japanese study only		Japanese and non-Japanese studies		Multinational study		Non-Japanese study only		No pediatric study		Total number of studies
All products^a^	31	18.1%	36	21.1%	46	26.9%	25	14.6%	33	19.3%	171
1. A confirmatory study was completed only in Japanese participants	10	100.0%									10
2. Extrapolation from non-Japanese children	3	3.2%	23	24.7%	41	44.1%	17	18.3%	9	9.7%	93
3. Extrapolation from Japanese adults and/or older children	14	14.0%	21	21.0%	33	33.0%	18	18.0%	14	14.0%	100
4. PMDA review without any of the above	6	21.4%	3	10.7%	1	3.6%	2	7.1%	16	57.1%	28

^a^Excluding overlaps between 2. and 3

#### Extrapolation from Non-Japanese Pediatric Studies

Non-Japanese pediatric studies (Table 6) were utilized for 93 products. Among them, pharmacokinetic similarity was discussed in 73.1% (68/93) of cases, whereas ethnic factor similarity was considered in 58.1% (54/93). Additionally, efficacy and safety similarity were evaluated for 57.9% (53/93) of the products. However, only 10.8% (10/93) of the products exhibited similar pharmacokinetic/pharmacodynamic (PK/PD) relationships. Regarding pharmacokinetics similarity, 46.2% (43/93) of products showed visually comparable pharmacokinetic parameters, whereas 32.3% (30/93) relied on predictions from population pharmacokinetic analysis models.

**Table 6:** Utilization of non-Japanese pediatric studies (N = 93)

	Discussion of extrapolation due to similarities in PK		Discussion of extrapolation due to similarities in PK/PD		Discussion of extrapolation due to similarities in ethnic factors		Discussion of extrapolation due to similarities in efficacy and safety		Total	
Number of products with extrapolation discussions	68	73.1%	10	10.8%	54	58.1%	53	57.0%	93	100.0%
Predictions using population analysis models	30	32.3%								
Visual comparisons of PK parameters	43	46.2%								
Predictions from non-Japanese children	60	64.5%	9	9.7%	39	41.9%	37	39.8%	93	100.0%
Predictions from Japanese adults and/or older children	28	30.1%	3	3.2%	29	31.2%	31	33.3%	60	64.5%
Predictions from other populations ^a^	1	1.1%	0	0.0%	0	0.0%	0	0.0%	1	1.1%
No extrapolation discussions	25	26.9%	83	89.2%	39	41.9%	40	43.0%	0	0.0%

^a^This review evaluated the efficacy of simoctocog alfa, a new active ingredient, for the control of bleeding tendency in patients with blood-clotting factor VIII deficiency. The approval was based on PK similarity of simoctocog alfa to an already approved coagulation factor VIII product and findings from pharmacology studies. These data supported the expectation that simoctocog alfa would be as effective as the approved product in children aged 2–12 years. Additionally, the similarities in ethnic factors between Japanese and non-Japanese were discussed in this PMDA review

*PD* pharmacodynamics, *PK* pharmacokinetics, *PMDA* Pharmaceuticals and Medical Devices Agency

#### Extrapolation from Japanese Studies in Adults and Older Children

Extrapolation from Japanese adults and/or older children was utilized in 100 products (Table 7). Pharmacokinetic similarities, PK/PD relationship similarities, disease similarities, efficacy, and safety similarities were evaluated.

**Table 7:** Utilization of Japanese studies in both adults and/or older children (N = 100)

	Discussion of extrapolation due to similarities in PK		Discussion of extrapolation due to similarities in PK/PD		Discussion of extrapolation due to similarities in ethnic factors		Discussion of extrapolation due to similarities in efficacy and safety		Total	
Number of products with extrapolation discussions	63	63.0%	10	10.0%	64	64.0%	62	62.0%	100	100.0%
Predictions using population analysis models	28	28.0%								
Visual comparisons of PK parameters	41	41.0%								
Predictions from non-Japanese children	41	41.0%	4	4.0%	26	26.0%	24	24.0%	61	61.0%
Predictions from Japanese adults and/or older children	43	43.0%	6	6.0%	52	52.0%	53	53.0%	100	100.0%
Predictions from other populations^a^	0	0.0%	2	2.0%	0	0.0%	0	0.0%	0	0.0%
No extrapolation discussions	37	37.0%	90	90.0%	36	36.0%	38	38.0%	0	0.0%

^a^For two products, similarities with non-pediatric populations were discussed. The first case was oblizumab, which added the indication of seasonal allergies, including in children, to already approved bronchial asthma. Population pharmacokinetic and clinical pharmacology studies demonstrated that serum IgE levels and body weight influenced pharmacokinetics and clinical pharmacology, whereas race, age, and disease-related effects had no significant impact. The mean serum-free IgE level was below the target of 25 ng/mL expected to be effective. Hence, the same dosage regimen for bronchial asthma was established for seasonal allergies. The other case was rituximab, approved for the treatment of organ transplant rejection, including in children. This product has already been approved for preventing organ transplant rejection. Given that rituximab reduces B-cell counts in target organs and the disappearance or decrease of DSA (Donner-specific antibody) and anti-HLA antibodies, the PMDA considered that the same pharmacokinetics profile could be achieved for this indication as in the previously approved indications

*PD* pharmacodynamics, *PK* pharmacokinetics, *PMDA* Pharmaceuticals and Medical Devices Agency

#### Products Approved Without Extrapolation

Regarding the 28 products (Table 5) where extrapolation was not discussed, the approval process included considerations of administrative incentives or other specific factors supporting drug approval. Nine products were recognized as medical necessities at the “Review Meeting of Unapproved Drugs and Off-Label Drugs with High Medical Needs.” Additionally, 11 products were approved based on public knowledge-based applications, and six products were designated as orphan drugs. Only one product lacked any of these attributes.

## Discussion

### Extraction of Analyzed Drugs and Analysis by Background

Pediatric indications represented 27.5% (172/625) of all approved products, ranging from 20 to 30% annually (Fig. 2), similar to PMDA’s reported percentage [1], confirming the validity of our data extraction. The low approval rate highlights a persistent gap between pediatric and adults. This is still a serious issue. Among 171 pediatric-approved products, 24.0% (41/171) included pediatric-specific doses, administration routes, and dosage forms (Fig. 3b). Once approved for adults, obtaining pediatric approval is difficult. Even if other countries permit adult formulations for pediatric use, Japan often requires pediatric-specific formulations [14], likely due to high national expectations for convenience and satisfaction, increasing costs and discouraging companies from seeking pediatric indications.

In Japan, intractable diseases are defined by the “Guidelines for Measures against Intractable Diseases” (1972) [15, 16], with 341 diseases designated as intractable[17]. In our analysis, 44 products were designated. The number of patients with intractable diseases was generally small, and 72.7% of them were also designated orphan drugs (Table 8). Among the identified 44 interactable disease drugs, 43.2% (19/44) were based on multinational studies (Fig. 6d). As noted in Sect."Patient Studies Included in PMDA Reviews", conducting a confirmatory P3 study solely in Japan is difficult. Even with limited patient numbers, multinational studies increase the number of patients to obtain statistical significance in P3 studies. Therefore, Japan to join the multinational study is important to be considered.

**Table 8:** Approved products (N = 44) for designated intractable diseases

Generic name	Indication	Designated intractable disease number	Administrative category
Risdiplam	Spinal muscular atrophy	3	Orphan drug
Nusinersen sodium	Spinal muscular atrophy without manifestation but onset predicted by genetic testing	3	Orphan drug
Eculizumab (genetic recombinant)	Generalized myasthenia gravis (only for patients with symptoms not controlled by high-dose intravenous immunoglobulin therapy or plasmapheresis)	11	Orphan drug
Satralizumab (genetic recombinant)	Prevention of relapse of neuromyelitis optica spectrum disorders (including neuromyelitis optica)	13	Orphan drug
Cerliponase alfa (genetic recombinant)	Ceroid lipofuscinosis type II	19	Orphan drug
Idursulfase beta (genetic recombinant)	Mucopolysaccharidosis type II	19	Orphan drug
Pabinafusp alfa (genetic recombinant)	Mucopolysaccharidosis type II	19	Orphan drug Pioneer drug designation system Sakigake
Avalglucosidase alfa (genetic recombinant)	Pompe disease	19	Orphan drug
Vestronidase alfa (genetic recombinant)	Mucopolysaccharidosis type VII	19	Orphan drug Pre-reviewed in “Review Meeting of Unapproved Drugs and Off-Label Drugs with High Medical Needs”
Olipudase alfa (genetic recombinant)	Acid sphingomyelinase deficiency	19	Orphan drug Pioneer drug designation system Sakigake
Migarastat hydrochloride	Fabry disease with GLA variant responsive to migarastat	19	Orphan drug
Selumetinib sulfate	Plexiform neurofibroma in neurofibromatosis type 1	34	Orphan drug
Secukinumab (genetic recombinant)	Psoriasis vulgaris, psoriatic arthritis, and pustular psoriasis (generalized)	37	None
Belimumab (genetic recombinant)	Systemic lupus erythematosus inadequately responsive to existing treatments	49	None
Rituximab (genetic recombinant)	Lupus nephritis inadequately responsive to conventional treatment	49	Public knowledge-based application after pre-evaluation
Anti-human thymocyte immunoglobulin, equine	Moderate-to-severe aplastic anemia	60	Orphan drug Pre-reviewed in “Review Meeting of Unapproved Drugs and Off-Label Drugs with High Medical Needs”
Eltrombopag olamine	Aplastic anemia	60	Orphan drug
Caplacizumab (genetic recombinant)	Acquired thrombotic thrombocytopenic purpura	63	Orphan drug
Defibrotide sodium	Hepatic sinusoidal obstruction syndrome (central veno-occlusive disease of the liver)	65	Orphan drug Pre-reviewed in “Review Meeting of Unapproved Drugs and Off-Label Drugs with High Medical Needs”
pH4-treated normal human immunoglobulin	Agammaglobulinemia or hypogammaglobulinemia	65	None
Berotralstat hydrochloride	Acute attacks of hereditary angioedema	65	Orphan drug Pioneer drug designation system Sakigake
Lanadelumab (genetic recombinant)	Acute attacks of hereditary angioedema	65	Orphan drug
Icatibant acetate	Acute attack of hereditary angioedema	65	Orphan drug
Human C1-inactivator	Acute attacks of hereditary angioedema	65	Orphan drug
Human normal immunoglobulin^a^	Agammaglobulinemia or hypogammaglobulinemia	65	None
Human normal immunoglobulin^b^	Agammaglobulinemia or hypogammaglobulinemia	65	None
Evinacumab (genetic recombinant)	Homozygous familial hypercholesterolemia	79	Orphan drug
Ambrisentan	Pulmonary arterial hypertension	86	Orphan drug
dalimumab (genetic recombinant)	Moderate or severe ulcerative colitis	97	None
Ravulizumab (genetic recombinant)	Atypical hemolytic uremic syndrome	109	None
Viltolarsen	Duchenne muscular dystrophy with confirmed deletion of the dystrophin gene that can be treated by exon 53 skipping	113	Orphan drug Pioneer drug designation system Sakigake Conditional early approval system
Fenfluramine hydrochloride	Concomitant therapy with antiepileptic drugs for epileptic seizures in patients with Dravet syndrome who do not respond adequately to other antiepileptic drugs	140	Orphan drug
Everolimus	Tuberous sclerosis	158	Orphan drug
Somatropin (genetic recombinant)	Body composition abnormalities in Prader-Willi syndrome	193	Orphan drug
Levothyroxine sodium hydrate	Myxedema coma Hypothyroidism (only if treatment with oral levothyroxine sodium is not appropriate)	235	Pre-reviewed in “Review Meeting of Unapproved Drugs and Off-Label Drugs with High Medical Needs”
Burosumab (genetic recombinant)	FGF23-related hypophosphatemic rickets and osteomalacia	238	Orphan drug
Givosiran sodium	Acute hepatic porphyria	254	Orphan drug
Vosoritide (genetic recombinant)	Achondroplasia without epiphyseal closure	276	Orphan drug
Sirolimus	Refractory lymphatic diseases (lymphangioma, lymphangiomatosis, Goham’s disease, lymphangiectasia)	277	Orphan drug
Albutrepenonacog alfa (genetic recombinant)	Inhibition of bleeding tendency in patients with blood coagulation factor IX deficiency	288	None
Simoctocog alfa (genetic recombinant)	Inhibition of bleeding tendency in patients with blood coagulation factor VIII deficiency	288	None
Concizumab (genetic recombinant)	Inhibition of bleeding tendency in congenital hemophilia patients with inhibitors of coagulation factors VIII or IX	288	Orphan drug
Efanesoctocog alfa (genetic recombinant)	Inhibition of bleeding tendency in patients with blood coagulation factor VIII deficiency	288	None
Lonafarnib	Hutchinson-Gilford-Progeria syndrome and processing-deficient progeroid laminopathy	333	Orphan drug

^a^Approval for Hizentra subcutaneous injection on June 26, 2023

^b^Approval for Cuvitru subcutaneous injection on September 25, 2023

Among 23 pediatric cancer drugs (Table 9), many were designated orphan drugs. Of these, 34.8% (8/23) were approved without pediatric studies (Fig. 6e), all based on the public knowledge-based applications by approval of “Review meeting of unapproved drugs and off-label drugs with a high medical need”.

**Table 9:** Approved products (N = 23) for pediatric cancer

Generic name	Indication	Administrative category
Entrectinib	*NTRK* fusion-positive advanced or recurrent solid tumors	Orphan drug Pioneer drug designation system Sakigake
Brentuximab vedotin (genetic recombinant)	CD30-positive peripheral T-cell lymphoma Relapsed or refractory CD30-positive Hodgkin’s lymphoma and peripheral T-cell lymphoma	Orphan drug
Alectinib hydrochloride	Relapsed or refractory *ALK* fusion-positive anaplastic large cell lymphoma	Orphan drug
Recombinant adsorbed 9-valent human papillomavirus virus-like particle vaccine (yeast origin)	Prevention of the following diseases caused by infection with human papillomavirus types 6, 11, 16, 18, 31, 33, 45, 52, and 58: Cervical cancer (squamous cell carcinoma and adenocarcinoma) and its precursor lesions (cervical intraepithelial neoplasia 1, 2, and 3 and intraepithelial adenocarcinoma) Vulvar intraepithelial neoplasia 1, 2, and 3 and vaginal intraepithelial neoplasia 1, 2, and 3 Condyloma acuminatum	None
Cytarabine	Acute leukemia (including cases of acute conversion of erythroleukemia and chronic myelogenous leukemia)	Public knowledge-based application
Recombinant adsorbed quadrivalent human papillomavirus virus-like particle vaccine (yeast origin)	Prevention of the following diseases caused by infection with human papillomavirus types 6, 11, and 16: Anal cancer (squamous cell carcinoma) and its precursor lesions (anal intraepithelial neoplasia 1, 2, and 3)	None
Daunorubicin hydrochloride	Acute leukemia (including acute transformation of chronic myelogenous leukemia)	Public knowledge-based application
Larotrectinib sulfate	*NTRK* fusion-positive advanced or recurrent solid tumors	Orphan drug
Dinutuximab (genetic recombinant)	Neuroblastoma after high-dose chemotherapy	Orphan drug
Filgrastim (genetic recombinant)	Enhancement of antitumor effects of dinutuximab (genetic recombinant) against neuroblastoma	Priority review
Teceleukin (genetic recombinant)	Enhancement of antitumor effects of dinutuximab (genetic recombinant) against neuroblastoma	Priority review
Busulfan	Pre-treatment for allogeneic HSCT (hematopoietic stem cell transplantation), pre-treatment for autologous HSCT in Ewing’s sarcoma family tumors and neuroblastoma	Public knowledge-based application after pre-evaluation Priority review Review Meeting of Unapproved Drugs and Off-Label Drugs with High Medical Needs
Nivolumab (genetic recombinant)	Relapsed or refractory classic Hodgkin lymphoma	Orphan drug
Selpercatinib	Unresectable, *RET* fusion gene-positive thyroid cancer Unresectable, *RET* gene mutation-positive medullary thyroid cancer	Orphan drug
Brentuximab vedotin (genetic recombinant)	Untreated CD30-positive Hodgkin lymphoma	Orphan drug
Lenograstim (genetic recombinant)	Combination therapy with antineoplastic agents for relapsed or refractory acute myeloid leukemia	Public knowledge-based application after pre-evaluation Priority review Review Meeting of Unapproved Drugs and Off-Label Drugs with High Medical Needs
Filgrastim (genetic recombinant)	Combination therapy with antineoplastic agents for relapsed or refractory acute myeloid leukemia	Public knowledge-based application after pre-evaluation Priority review Review Meeting of Unapproved Drugs and Off-Label Drugs with High Medical Needs
Fludarabine phosphate	Relapsed or refractory acute myelogenous leukemia	Public knowledge-based application after pre-evaluation Priority review Review Meeting of Unapproved Drugs and Off-Label Drugs with High Medical Needs
Cytarabine^a^	Acute leukemia	Public knowledge-based application Priority review
Cytarabine^b^	Acute leukemia	Public knowledge-based application Priority review
Pegaspargase	Acute lymphocytic leukemia, malignant lymphoma	None
Dabrafenib mesylate	Advanced or relapsed solid tumors (excluding colorectal cancer) with *BRAF* mutations that are refractory to standard treatment Relapsed or refractory hairy cell leukemia with *BRAF* mutations	Orphan drug
Trametinib dimethyl sulfoxide adduct	Advanced or recurrent solid tumors (excluding colorectal cancer) with *BRAF* mutations that are refractory to standard treatment	Orphan drug

^a^Approval for Cylocide (normal preparation) injection on 25 May 2023

^b^Approval for Cylocide N (high-dose preparation) injection on 25 May 2023

For children aged 12 years and older, most of the products analyzed had been approved (Fig. 4). In 2020, a notification, “Points to consider in the clinical assessment of adults and evaluable children (children aged 10 or 12 years or older),” was issued [18], allowing children aged ≥ 10 years to be included in adult clinical trials if the disease, study endpoint, dosage, and formulations were similar. In our analysis, 26 products evaluations enrolled pediatric patients in adult confirmatory clinical studies. However, approval rates decreased with age; only 25.1% for neonates (< 4 weeks) and 22.2% for Teishussei taijuu-ji (preterm newborn infants). Younger children require age-matched formulations. Generally, drug formulations for children aim to match adult exposure; however, physiological differences can lead to unexpected exposure differences [19, 20]. In addition to solubility issues, a palatable oral formulation may be necessary [3, 21]. Infants and neonates differ significantly from adults [22], requiring tailored formulations. Moreover, the smaller patient population renders profitability and obtaining informed consent in clinical studies difficult [23–25], increasing difficulties in approval with decreasing patient age. Such problems persist in Western countries mandating pediatric drug development, highlighting the need for benefits to the companies.

### Administrative Incentives

**Fig. 5:**
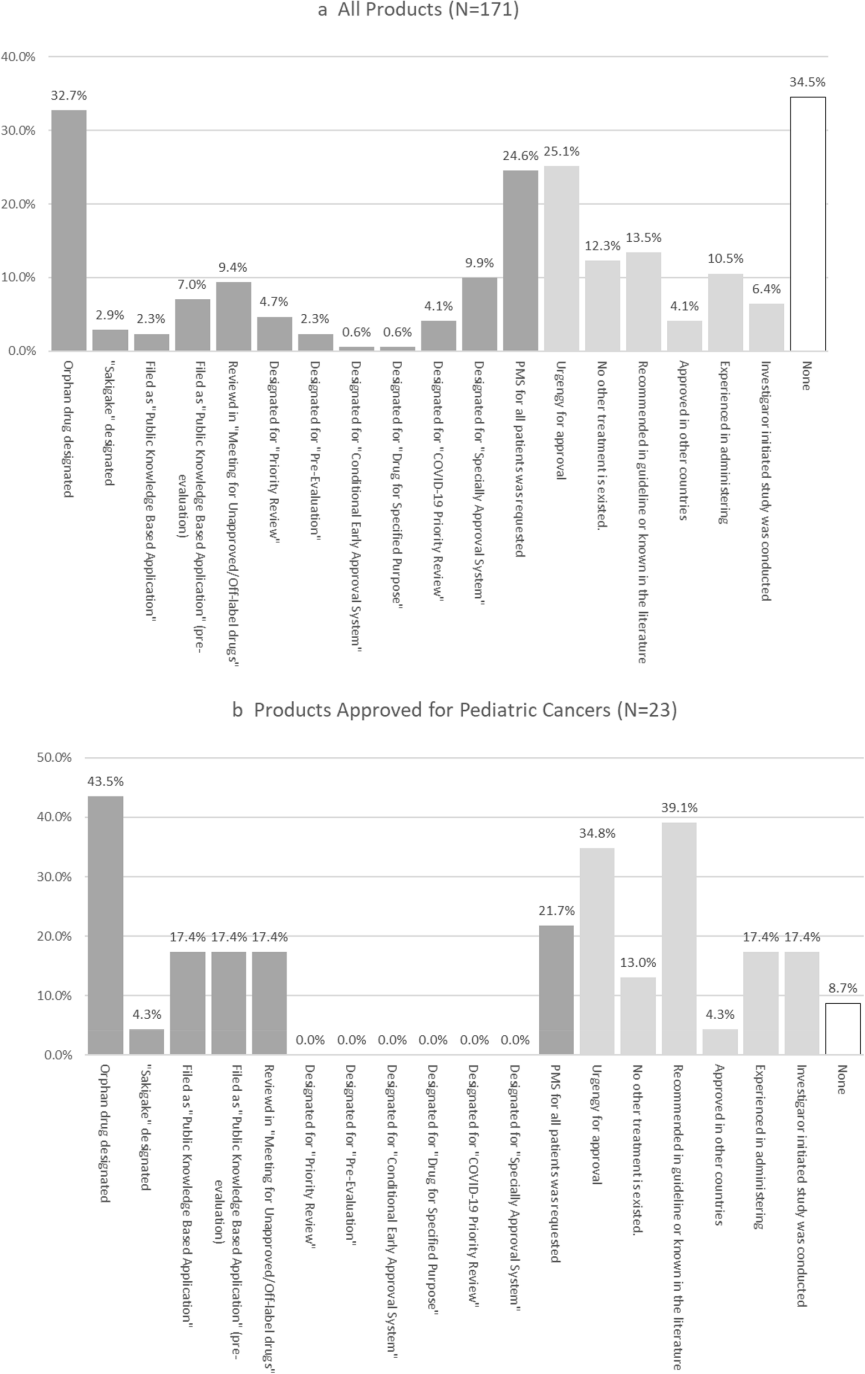
Administrative incentives and other issues considered during the review process. Dark-shaded bars represent items with administrative incentives. Lighter shaded bars indicate items in the review report that support approval, excluding administrative incentives. An overlap was noted in counting approved products. The right-most category, “None,” indicates items without any of the listed elements.

Japan offers various administrative incentives for drug development. Among the analyzed items, orphan drugs were most common, accounting for 32.7% (Fig. 5a). To receive orphan drug designation, the following conditions must be met:

- The patient population is small, approximately ≤ 50,000 or ≤ 1/1,000 of the population (~ 120,000) for designated intractable diseases.
- There is a medical need, meaning the disease is serious, and the drug is useful for treatment.
- Domestic drug development systems are in place, and a development plan is established.

For designated intractable diseases, justification is based on patient numbers and medical necessity. The Ministry of Health, Labor, and Welfare (MHLW) manages the process, with acceptability determined through interviews with the “Pharmaceutical Affairs and Food Sanitation Council,” an advisory body [26, 27]. Once designated, benefits include grants from the National Institute of Biomedical Innovation, Health and Nutrition (NIBIO), prioritization in PMDA consultations and approval reviews, and extended exclusive marketing periods. Among 56 orphan products in the analysis, 16 underwent confirmatory studies in non-Japanese or multinational studies. In some cases, confirmatory studies are not required, as authorities recognize recruitment challenges. However, for products where Japan participated in multinational trials, confirmatory study results were submitted for 8 of 56 products. Recently, confirmatory studies may be required for orphan drugs, with expectations for Japanese participation in multinational trials. Data consistency between Japanese and total (including non-Japanese) populations is required, but a large number of Japanese patients is unnecessary.

The"Review Meeting of Unapproved Drugs and Off-Label Drugs with High Medical Needs"evaluates drugs requested by academic societies that are approved in Europe and the USA but not in Japan. This body recognized 9.4% of products (Fig. 5a) as necessary. In such cases, a development request is made to the development company, or a company is recruited to develop the product [28]. This body also assesses whether a public knowledge-based application is appropriate, allowing approval in Japan without conducting all or part of the studies locally if the product is approved in another country with an equivalent regulatory system. A public knowledge-based application can be submitted for products validated at the review meeting. As a result, 7.0% of products (Fig. 5a, “Filed as ‘Public Knowledge-Based Application’ (pre-evaluation)”) can be applied through this system. CTDs were not submitted for these applications; instead, request forms detailing approval status in foreign countries, usage data, and relevant guidelines or medical references were provided.

The “Special approval” under the “Pharmaceutical and Medical Device Act” applied to 9.9% of products (Fig. 5a) [29] an early approval system based on the opinion of the “Pharmaceutical Affairs and Food Sanitation Council,” is for products urgently needed to prevent disease spread with serious public health impacts when no alternatives exist. The product must be marketed in a country with an equivalent approval system. This approval process applied to vaccines for the 2010 influenza pandemic and SARS-CoV- 2 treatments. All 17 products in Fig. 5a were SARS-CoV- 2 vaccines or therapeutics. In 2022, the "Emergency Approval" system was introduced. While “Special Approval” requires confirmation of drug efficacy through marketing authorization in another country with an approval system equal to Japan, the “Emergency Approval System” only requires presumed efficacy for drug approval. Specifically, the “Emergency Approval System” is designed to ease approval conditions in Japan for new products under development in the USA and EU.

Additionally, Japan has a conditional early approval system, allowing approval without confirmatory clinical studies, under conditions such as mandatory post-marketing survey of efficacy and safety, targeting a serious disease with no effective therapy, a small patient population, and difficulties or delays in conducting clinical studies in Japan. In our analysis, viltolarsen (indication for Duchenne muscular dystrophy with deletion of the dystrophin-encoding gene that can be treated by exon 53 skipping, approved on March 25, 2002) is the only product for which this system was applied.

Moreover, for 24.6% of all products, companies were required to include all patients using the marketed products in the post-marketing survey (Fig. 5a), and products were approved without sufficient Japanese patients in clinical trials. Those cases are similar to the conditional early approval system. Even if confirmatory studies have not been conducted, approval can be granted if the efficacy and safety can be predicted. The condition is to collect detailed data from post-marketing surveys via close monitoring of all patients who received the product.

Figure 5a includes five products approved under the “Pioneer drug designation system Sakigake.” The system aims to provide patients with the most advanced therapeutic products developed in Japan earlier than in other countries. This system ensures priority handling during PMDA consultations and reviews for marketing authorization. Moreover, it includes consultation on manufacturing and distribution to facilitate rapid product launch [30].

### Patient Studies Included in PMDA Reviews

**Fig. 6:**
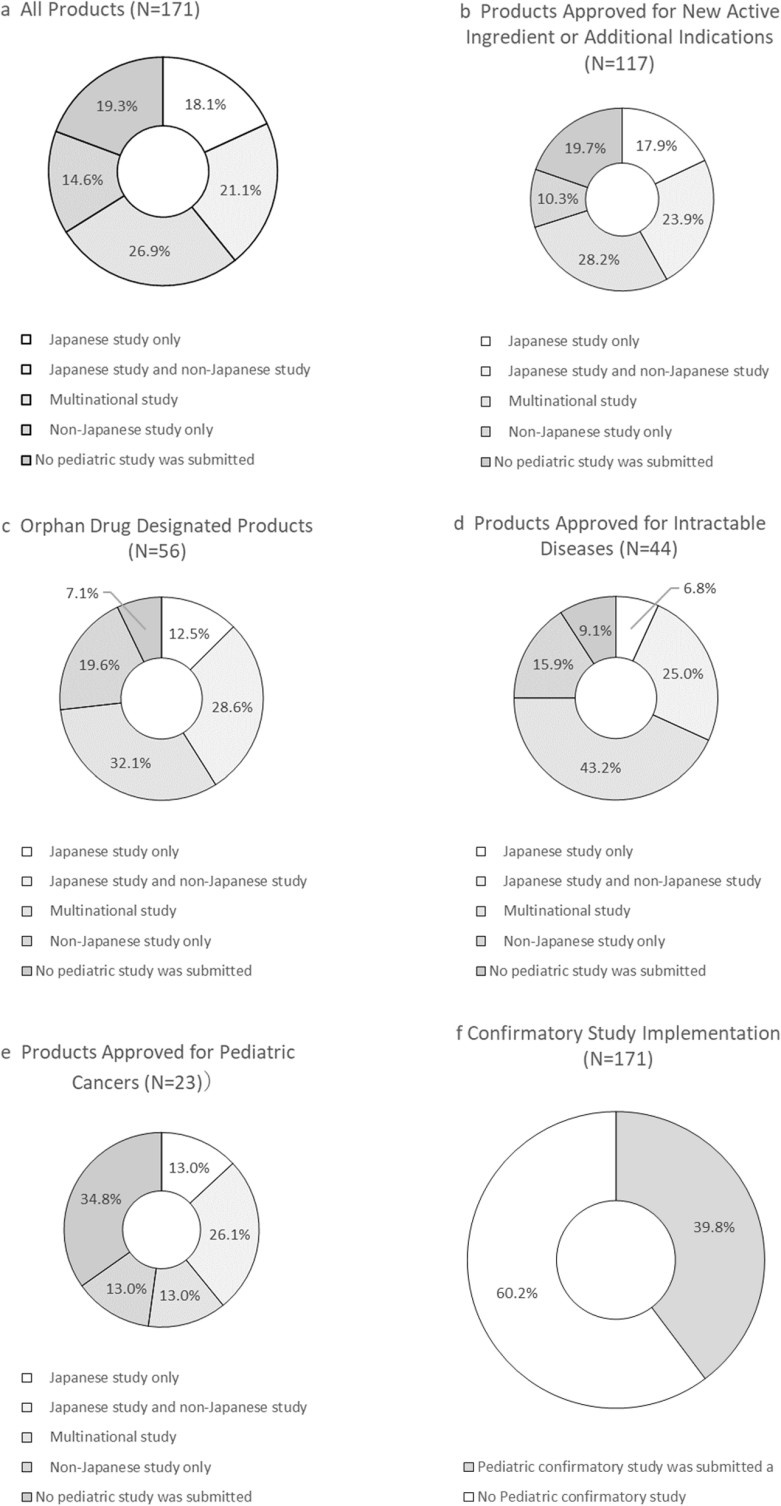
Type of submitted pediatric clinical studies based on the backgrounds of approved products. (a) The percentages of products for which clinical trials were conducted exclusively in children or both children and adults in confirmatory studies

Only 18.1% of products were approved based solely on Japanese studies (Fig. 6a), and only 10 were statistically confirmed exclusively for pediatric subjects (Table 3). This suggests that large-scale confirmatory studies for pediatric patients are limited to specific disease areas in Japan. The pediatric population is smaller than the adult population, and Japan faces declining birthrates and an aging society. Additionally, surveys of pharmaceutical companies, drug discovery ventures, and medical institutions in Japan have reported a shortage of medical centers capable of conducting pediatric clinical studies and difficulties in recruiting pediatric patients [14, 31].

Many products have been approved using extrapolations from non-Japanese children and Japanese adults and/or older children, following ICH E5 (Ethnic Factors in the Acceptability of Foreign Clinical Data) and ICH E11 A (Pediatric Extrapolation). Extrapolation involves using clinical trial data from one country or region to support approval in another, aiming to reduce redundant trials and expedite patient access to beneficial pharmaceuticals. Effective extrapolation requires a thorough assessment of ethnic factors, as outlined in ICH E5. Beyond geographic extrapolation, this concept now extends to extrapolation from adults to children and from older to younger children, as described in ICH E11 A. The following factors in the ICH E5 are relevant to extrapolability:

- Minimal ethnic factors
- Similar dose response, safety, and efficacy between the new and original regions
- Comparable safety and efficacy despite different doses, if supported by pharmacokinetic or pharmacodynamic studies, allow dose adjustments to justify the extrapolation of foreign data.

**Fig. 7:**
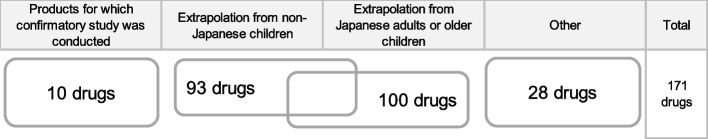
Products with statistical confirmatory studies and extrapolation from other patient groups

**Fig. 8:**
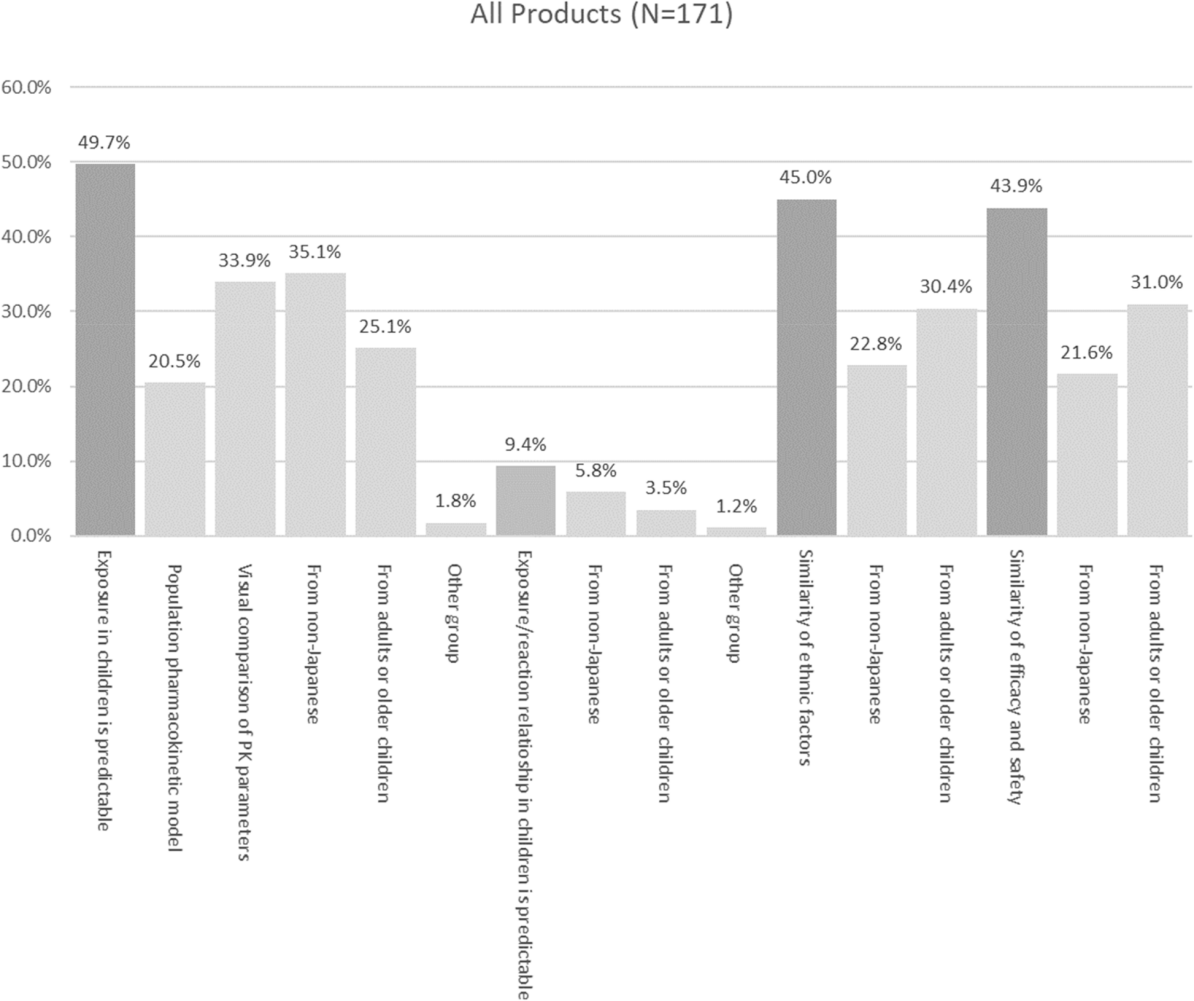
Rates of products with extrapolation from non-Japanese data or Japanese adults and/or older children

In this study, 93 of 171 products were evaluated for similarities in at least one of four aspects: pharmacokinetics, PK/PD, ethnic factors, or clinical study results in non-Japanese children. Additionally, similarities with Japanese adults and/or older children were examined for 100 products (Figs. 7 and 8).

While pharmacokinetic similarities were widely discussed for many products, mostly through visual comparisons of pharmacokinetic parameters, PK/PD similarity discussions were limited to only a few products, likely due to the scarcity of suitable biomarkers or clinical pharmacology endpoints for most products. Similarity assessments using population analysis models have been proposed; however, many products have limited PK data, resulting in insufficient data for robust population pharmacokinetic analyses.

Ethnic similarity is also an important consideration but was discussed in only 45.0% of products. In some cases, ethnic factors may have already been evaluated in the review of another product for the same disease. Extrapolation based on similarities in efficacy and safety was considered in 43.9% of products. Elements from these four aspects are discussed in each application dossier based on the target disease.

The U.S. FDA published the guidance *Exposure–Response Relationships—Study Design, Data Analysis, and Regulatory Applications* in 2003 [32]. Extrapolation possibilities have been evaluated on a disease-by-disease basis, with specific guidance available for some disease areas [33, 34]. While no such guidance exists in Japan, European and U.S. guidelines are likely referenced during reviews in Japan.

Multinational studies play a key role in similarity discussions. Among the 68 products with submitted pediatric confirmatory studies, 27 involved multinational pediatric studies including Japan. According to the *Guidance for Japanese Participation in Global Clinical Trials* [35], statistical power is not required to detect significant differences within the Japanese subgroup. Instead, sample sizes should ensure consistency between the overall study population and the Japanese subpopulation. Generally, including 15–20% Japanese patients is recommended for statistical confirmation, but the specific sample size should be discussed with the PMDA. Even with a limited number of Japanese patients, a multinational study can serve as a confirmatory study, making Japanese participation important when designing multinational trials.

The concept of extrapolating data from adults and/or older children is partially introduced in ICH E11 A. While the general extrapolation principles in ICH E11 A are similar to those in ICH E5, similarities in ICH E5 can be replaced with disease similarities in adults and/or older children. Analyses in this study followed this framework (Table 7), and extrapolation from Japanese adults and/or older children was discussed for many products.

Twenty-five products were approved without extrapolation from foreign children or Japanese adults. All were based on clinical trials with a small number of patients and were granted approval through administrative handling due to the urgent need for treatment, the absence of alternatives, and the severity of the target disease.

## Summary

Statistical analyses are not always required when patient numbers are small, such as in orphan diseases or urgent approvals for refractory diseases. In high-urgency cases, a public knowledge-based application may be accepted without a clinical trial if a patient is refractory or critically ill with no alternative treatment. While efficacy and safety assessments may be limited during PMDA review, early access to therapeutic drugs is prioritized. If a sufficient number of Japanese patients cannot be recruited before approval, post-marketing data collection for all patients helps acquire additional evidence.

In Japan’s pediatric drug development, extrapolation—utilizing data from other patient groups—is common. When extrapolating from non-Japanese children, participation in multinational studies is the standard approach. In such cases, demonstrating data consistency between Japanese patients and the overall study population is essential. If Japan cannot participate in multinational trials, Japanese pediatric patients should be enrolled in other studies whenever possible to facilitate similarity discussions based on PK, PK/PD, ethnic factors, efficacy, and safety. For diseases with few patients, rendering Japanese recruitment difficult, consulting regulatory authorities (PMDA) in advance regarding sample size requirements is crucial. It is also advisable to ensure PMDA understands that recruiting a sufficient number of Japanese patients may be challenging due to disease characteristics.

Indications for children aged ≥ 12 years may be approved based on clinical trial results conducted alongside adults. If Japan participates in a multinational clinical study, the required number of Japanese patients may be reduced. Therefore, ensuring Japan’s participation during study planning is essential.

## Conclusions

This study is the first to clarify the extrapolation of non-Japanese data to Japan using ICH E5 in the approval process for Japanese pediatric drugs, as well as the extrapolation from adults to children and from older to younger children using ICH E11 A.

Furthermore, this study is the first to highlight the use of administrative incentives by Japanese regulatory authorities. Regulatory authorities may permit preferential measures if conditions are met. Since PMDA can be consulted on the validity of the clinical data package, early consultation is crucial. It facilitates pediatric drug approval.

The results of this study provide development strategies for pharmaceutical industries seeking pediatric drug indications in Japan, groups planning investigator-initiated studies (IIS). We believe this can help promote pediatric drug development in Japan.

As the next step, introducing specific discussion points for the approval of drugs for intractable diseases and pediatric cancers will help accelerate the approval process for these challenging conditions.

## Limitation

A key limitation is that detailed discussions between PMDA and applicants during the approval review process, including query/answer interaction and meeting minutes, are not publicly disclosed. While some of this information is reflected in the PMDA review report, it is not comprehensive. Hence, our research is limited to the information available in the PMDA report.

## Data Availability

The research data can be provided based on the request.

## Abbreviations

CTD: Common Technical Documents
ICD- 10: International Classification of Disease
ICH: International Council for Harmonization of Technical Requirements for Pharmaceuticals for Human Use
MHLW: The Ministry of Health, Labor and Welfare
NIBIO: National Institute of Biomedical Innovation, Health and Nutrition
PD: Pharmacodynamics
PK: Pharmacokinetics
PMDA: The Pharmaceuticals and Medical Devices Agency
